# Hybrid 2D MoS_2_–Copper Benzene-1,3,5-Tricarboxylate Framework for Room-Temperature Gas Sensing: Toward Trace Ammonia Detection for Hydrogen-Carrier Safety

**DOI:** 10.3390/s26217082

**Published:** 2026-11-09

**Authors:** Tran Thanh Tung, Anh Tuan Trong Tran, Md Abdul Jalil, Tran Van Khai, Thuy Ai Huyen Le, Duc Thuan Lao, Mickael Castro, Jean-Francois Feller, Nguyen Xuan Sang, Dusan Losic

**Affiliations:** 1 School of Chemical Engineering, Adelaide University, Adelaide, SA 5005, Australia; trongtuananh.tran@adelaide.edu.au (A.T.T.T.); mdabdul.jalil@adelaide.edu.au (M.A.J.); 2 VNU-HCM Key Laboratory for Material Technologies, Ho Chi Minh City University of Technology (HCMUT), 268 Ly Thuong Kiet Street, Dien Hong Ward, Ho Chi Minh City 70000, Vietnam; tvkhai1509@hcmut.edu.vn; 3 Vietnam National University—Ho Chi Minh City (VNU-HCM), Quater 33, Linh Xuan Ward, Ho Chi Minh City 71308, Vietnam; 4 Center for Life Science Research, Ho Chi Minh City Open University, 97 Vo Van Tan, Xuan Hoa Ward, Ho Chi Minh City 70000, Vietnam; thuy.lha@ou.edu.vn (T.A.H.L.); thuan.ld@ou.edu.vn (D.T.L.); 5 Smart Plastics Group, IRDL CNRS 6027, University of South Brittany (UBS), 56321 Lorient, France; mickael.castro@univ-ubs.fr (M.C.); jean-francois.feller@univ-ubs.fr (J.-F.F.); 6 Atomic Molecular and Optical Physics Research Group, Institute for Advanced Study in Technology, Ton Duc Thang University, Ho Chi Minh City 72912, Vietnam; nguyenxuansang@tdtu.edu.vn; 7 Faculty of Electrical and Electronics Engineering, Ton Duc Thang University, Ho Chi Minh City 70000, Vietnam

**Keywords:** 2D material, MoS_2_, Cu-based MOF, VOC sensor, hybrid material

## Abstract

**Highlights:**

**What are the main findings?**

• CuBTC–MoS_2_ exhibited superior conductivity and sensing performance compared with ZIF-8–MoS_2_ and UiO-66–MoS_2_.
• Sub-ppm NH_3_ detection was achieved with ~35% response at 0.8 ppm and rapid response/recovery at room temperature.
• Synergistic MOF adsorption and MoS_2_ transduction enable sensitive NH_3_ monitoring for ammonia-based hydrogen-carrier safety.

**What are the implications of the main findings?**

• Demonstrates a synergistic MOF–MoS_2_ strategy for developing sensitive, selective, and low-power room-temperature chemoresistive gas sensors.
• Highlights CuBTC–MoS_2_ as a promising platform for sub-ppm NH_3_ leakage monitoring, supporting safer storage, transportation, and utilisation of ammonia as a hydrogen carrier.

**Abstract:**

Room-temperature detection of trace ammonia (NH_3_) is essential for hydrogen-carrier safety, yet achieving high sensitivity and analyte discrimination remains challenging. Here, three metal–organic frameworks (CuBTC, ZIF-8, and UiO-66) were hybridised with two-dimensional MoS_2_ and comparatively evaluated for room-temperature chemoresistive sensing of NH_3_ and selected volatile organic compounds (VOCs). Among these hybrids, CuBTC–MoS_2_ exhibited the highest and most reproducible NH_3_ response, reaching approximately 35% at 0.8 ppm, with a calculated limit of detection (LOD) of 0.013 ppm (13 ppb). The sensor demonstrated a superior response toward NH_3_ than other investigated gases, including formaldehyde, especially at concentrations of 0.8–10 ppm, whereas formaldehyde produced a stronger response above approximately 17 ppm. Rapid response and recovery times of 6 s and 9 s, respectively, were achieved. The enhanced NH_3_ response is attributed to favourable adsorption within CuBTC and interfacial charge modulation in the CuBTC–MoS_2_ hybrid. These findings highlight the potential of CuBTC–MoS_2_ for trace NH_3_ monitoring in hydrogen-carrier systems while identifying concentration-dependent cross-sensitivity as a consideration for practical applications.

## 1. Introduction

The rapid development of the hydrogen economy has created increasing demand for safe, efficient, and scalable technologies for hydrogen storage, transportation, and utilisation [1,2,3]. Although hydrogen (H_2_) is an attractive clean energy carrier, its low volumetric energy density and demanding storage conditions remain major barriers to large-scale distribution. Consequently, hydrogen-rich chemical carriers have attracted considerable attention as alternative approaches for storing and transporting hydrogen. Among them, ammonia (NH_3_) is particularly promising because of its high hydrogen content, established production and transportation infrastructure, and ability to be stored and transported more readily than compressed or liquefied hydrogen. Despite these advantages, the increasing use of ammonia as a hydrogen carrier introduces significant safety challenges throughout its production, storage, transportation, distribution, and utilisation [4,5]. NH_3_ is a toxic and corrosive gas, and accidental leakage can pose serious risks to workers, surrounding communities, and the environment. Exposure can cause irritation of the eyes, skin, and respiratory tract, while higher concentrations can lead to severe respiratory injury and other adverse health effects [1,2,3,4]. Accordingly, reliable monitoring and early detection of NH_3_ leakage are essential for the safe implementation of ammonia-based hydrogen technologies. Safety requirements are particularly demanding for distributed hydrogen-energy systems, where sensors should provide high sensitivity at low NH_3_ concentrations, rapid response and recovery, good selectivity, long-term stability, and reliable operation under varying environmental conditions. A range of sensing technologies has therefore been investigated for NH_3_ detection, among which chemoresistive sensors are particularly attractive because of their simple device architecture, low cost, ease of miniaturisation, and compatibility with portable and wireless monitoring systems [5,6,7]. Conventional chemoresistive gas sensors predominantly employ semiconducting metal oxides. Despite considerable progress and widespread commercial use, many metal-oxide sensors require elevated operating temperatures, resulting in increased power consumption and limiting their integration into low-power and wearable or distributed sensing platforms [8,9,10]. Furthermore, insufficient selectivity, humidity interference, response/recovery characteristics, and long-term stability remain important challenges, particularly when NH_3_ must be distinguished from other gases and volatile organic compounds (VOCs) present in complex operating environments. These limitations highlight the need for highly sensitive, selective, rapid, and stable NH_3_ sensors capable of operating at room temperature. Such sensors should not only detect low concentrations of NH_3_ but also discriminate NH_3_ from potentially interfering gases and vapours, including methanol, ethanol, acetone, and formaldehyde. Room-temperature chemoresistive sensing is particularly attractive in this context because adsorption of target molecules directly modulates the electrical properties of the sensing material, enabling concentration-dependent detection without the continuous heating required by many conventional metal-oxide sensors [7,8]. Accordingly, engineering sensing materials that simultaneously provide strong NH_3_ adsorption, selective molecular recognition, efficient charge transport, and environmental stability is critical for developing next-generation ammonia sensors toward safe hydrogen-carrier technologies [9,10,11].

A wide range of sensing materials has been investigated for gas detection, including zero-dimensional (0D) nanoparticles (metal/alloy nanoparticles and carbon nanomaterials), one-dimensional (1D) nanofibres, nanowires and nanotubes, and two-dimensional (2D) materials such as graphene derivatives, MXenes, and transition metal dichalcogenides (TMDs) [12,13,14,15]. Among these, 2D TMDs, particularly molybdenum disulfide (MoS_2_), molybdenum diselenide (MoSe_2_), and tungsten disulfide (WS_2_), have attracted significant attention for room-temperature gas sensing owing to their high surface-to-volume ratio, tuneable electronic structures, abundant surface-active sites, and strong gas adsorption capability [16,17,18]. MoS_2_ is especially attractive because of its polymorphic nature, where semiconducting 2H and metallic 1T phases can coexist, enabling modulation of electrical conductivity, charge transport, and surface reactivity [19]. These properties make MoS_2_ a promising platform for low-power chemical and gas sensing applications, including the detection of ammonia leakage in hydrogen-carrier systems. However, pristine MoS_2_ often exhibits limited sensitivity and selectivity, particularly under complex operating environments where interfering gases and humidity are present [20,21]. Consequently, substantial research efforts have focused on enhancing its sensing performance through defect engineering, heteroatom doping, surface functionalisation, and the construction of hetero-structures and hybrid nanocomposites that improve adsorption, charge transfer, and molecular recognition toward target analytes [20,21].

Metal–organic frameworks (MOFs), the 2025 Chemistry Nobel Prize-recognized class of materials, represent another highly promising platform for gas sensing. Their exceptional structural diversity, high specific surface area, tunable pore size and chemistry, and abundant adsorption sites enable selective capture and molecular recognition of target gases [22,23]. These characteristics also make MOFs attractive functional components for integration with electrically conductive sensing materials [24,25,26,27]. In particular, coupling MOFs with 2D MoS_2_ provides a promising strategy for room-temperature NH_3_ detection, where the porous MOF can enhance gas adsorption and molecular selectivity, while MoS_2_ provides an efficient pathway for translating adsorption-induced interfacial charge transfer into a measurable electrical response. Such complementary functions offer opportunities to engineer sensitive, selective, and low-power NH_3_ sensors for monitoring leakage in ammonia-based hydrogen storage, transportation, and utilisation systems. However, MOFs vary substantially in metal nodes, organic linkers, pore structures, surface functionalities, and chemical stability, resulting in markedly different interactions with NH_3_ and other interfering gases or vapour. Consequently, it remains unclear which MOF chemistry provides the most favourable synergistic interaction with MoS_2_ for low-concentration NH_3_ detection, and how different MOF–MoS_2_ combinations influence sensitivity, selectivity, response kinetics, and environmental stability. Addressing this knowledge gap is therefore important for the rational design of MOF–MoS_2_ hybrid chemoresistive sensors for reliable room-temperature NH_3_ monitoring and hydrogen-carrier safety. In this study, three representative metal–organic frameworks (MOFs), namely ZIF-8 (comprising Zn^2+^ ions and 2-methylimidazolate linkers), UiO-66 (Zr_6_O_4_(OH)_4_ clusters and terephthalate linkers), and CuBTC (Cu^2+^ ions and benzene-1,3,5-tricarboxylate linkers), were systematically hybridized with two-dimensional (2D) MoS_2_ and comparatively evaluated for room-temperature chemoresistive gas sensing. This study addresses an important research gap, as previous investigations of related MOF-based sensing materials have predominantly focused on elevated operating temperatures (typically 200–400 °C), while their potential for room-temperature gas sensing remains relatively unexplored.

Their distinct metal nodes, linker chemistries, and pore environments provide a platform for examining how MOF composition influences molecular adsorption and sensing behaviour when coupled with MoS_2_. Accordingly, this study aims to comparatively investigate three representative MOF–MoS_2_ hybrids (CuBTC–MoS_2_, ZIF-8–MoS_2_, and UiO-66–MoS_2_) for room-temperature chemoresistive detection of NH_3_ and selected interfering gases and volatile organic compounds (VOCs). Particular emphasis is placed on evaluating sensing sensitivity, selectivity, response/recovery characteristics, and low-concentration NH_3_ detection relevant to hydrogen-carrier safety. By examining the influence of MOF chemistry on molecular adsorption and electrical signal transduction, this work seeks to establish structure–property relationships that can guide the rational design of MOF–TMD hybrid sensing materials for low-power NH_3_ monitoring in hydrogen-carrier storage, transportation, and utilisation.

## 2. Materials and Methods

### 2.1. Materials and Chemicals

Zinc carbonate basic ([ZnCO_3_]_2_·[Zn(OH)2]_3_, Sigma Aldrich, Adelaide, Australia), 2-methylimidazole (C_4_H_6_N_2_, Aldrich, 99%), zirconium chloride (ZrCl_4_, 99.5%; Sigma Aldrich), 1,4-benzenedicarboxylate (H2BDC, 98%; Sigma Aldrich), N,N-dime-thylformamide (HCON(CH_3_)_2_, DMF, 99.8%; Sigma-Aldrich), methanol (CH_3_OH, 99.5%, Chem-supply); all chemicals and solvents were purchased from commercial sources and used as received without further purification. MoS_2_ fine powder (99.9%) with particle size of ~2–5 μm was purchased from Sigma-Aldrich (Australia).

### 2.2. Preparation of MOFs and Their Hybrid with MoS_2_

**Figure 1:**
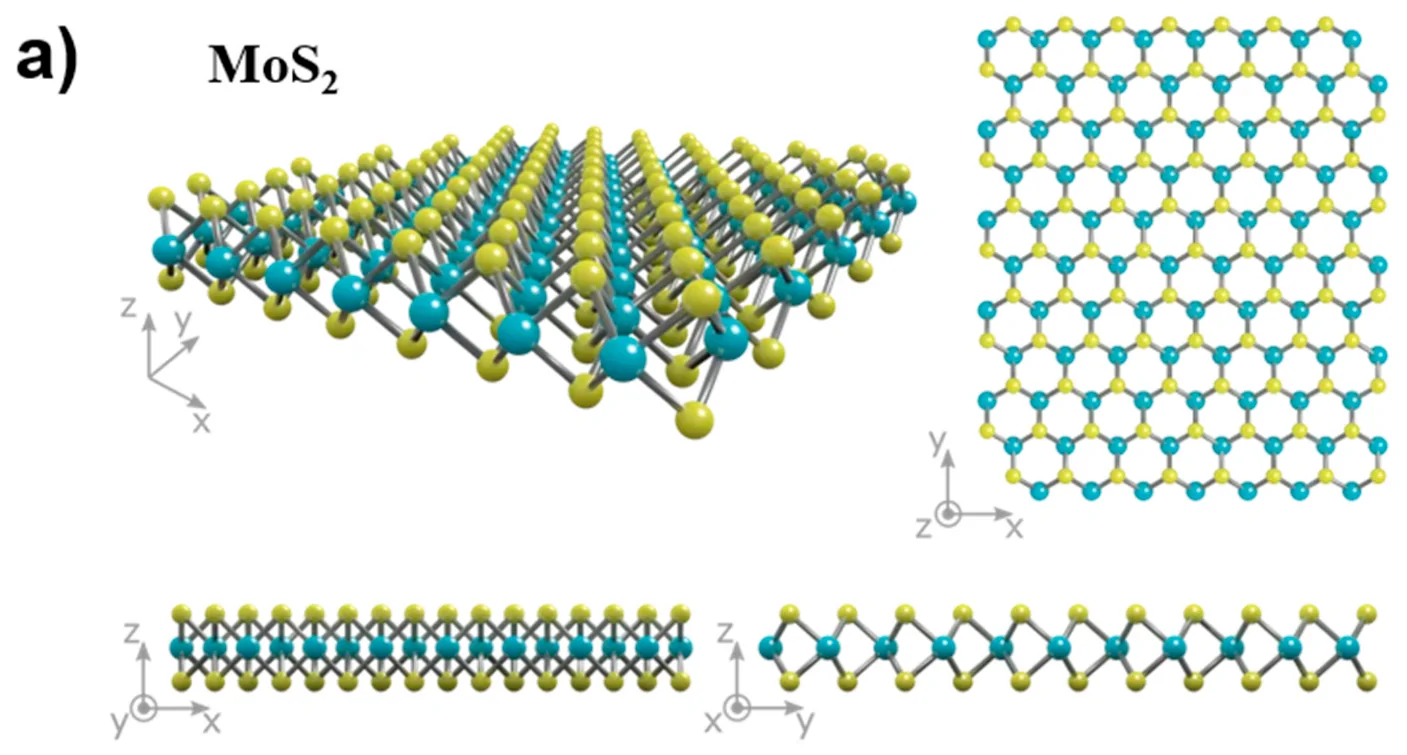

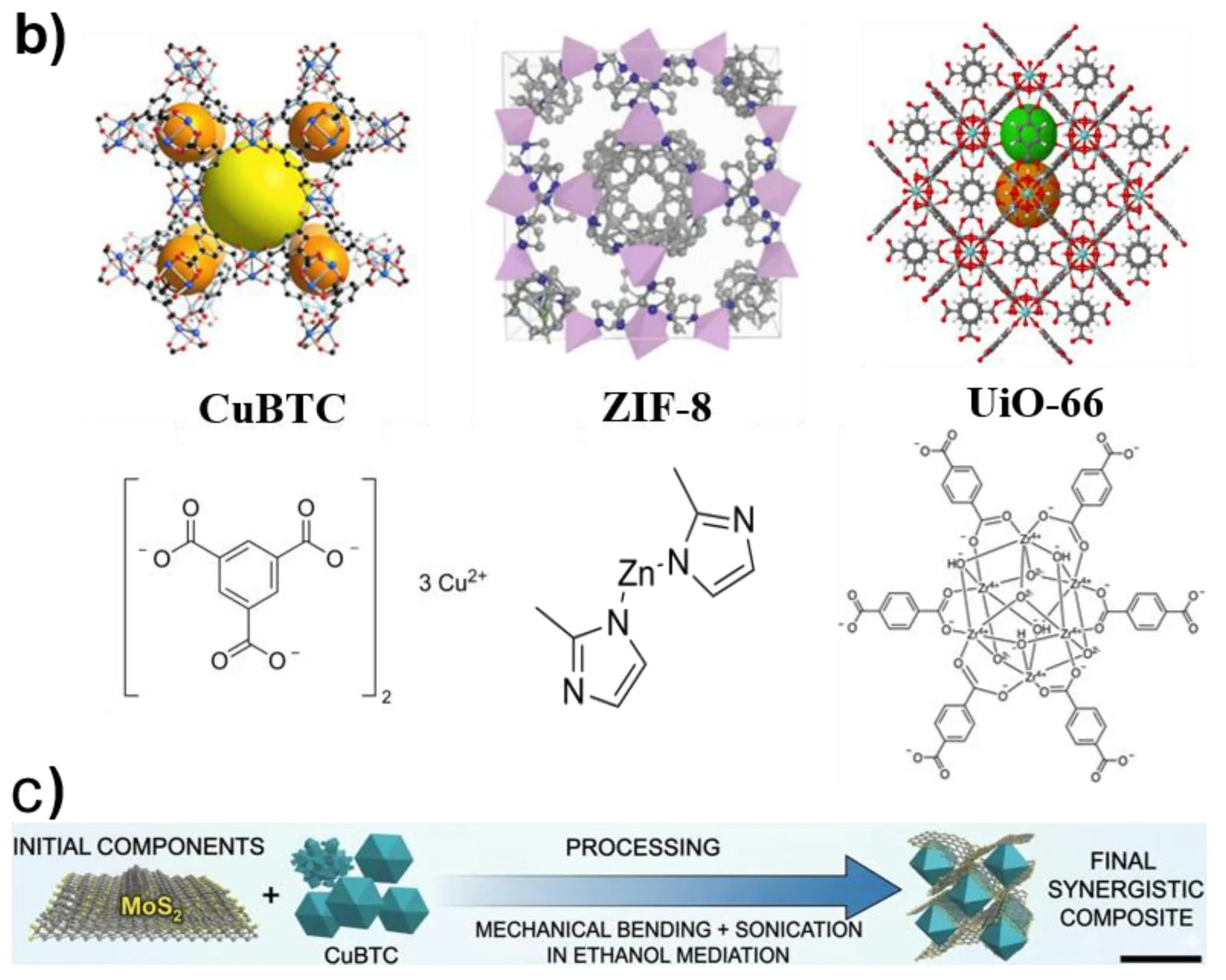
Illustrates the initial crystallographic profiles, molecular coordination environments, and the liquid-phase integration workflow utilized to construct the hybrid material. Panel (**a**) presents the pristine 2H-phase MoS_2_ structure across multiple crystallographic perspectives, defining the atomic positioning of the coordinating sulfur edges relative to the inner molybdenum planes. Panel (**b**) details the comparative three-dimensional porous cages and corresponding chemical building blocks of the chosen MOF candidates: CuBTC with its open copper nodes, ZIF-8 featuring zinc-imidazolate linkages, and UiO-66 composed of zirconium clusters. The processing flowchart (**c**) shows the targeted synthesis route, where concurrent mechanical bending and acoustic cavitation work together to break down and link the initial phases into a cohesive CuBTC-MoS_2_ network.

Figure 1 illustrates the crystal structure of MoS_2_ (a), MOF coordination structure (b), and formation of MoS_2_–MOF hybrids by mechanical blending (c). Zirconium 1,4-dicarboxybenzene framework (UiO-66), Zeolitic imidazolate framework-8 (ZIF-8), and copper benzene-1,3,5-tricarboxylate framework (CuBTC) were synthesised and purified following previously reported procedures [26]. Briefly, Cu-BTC was prepared by refluxing copper nitrate trihydrate (1.75 g) and 1,3,5-benzenetricarboxylic acid (0.84 g) in ethanol at 85 °C for 24 h, followed by filtration and washing with water and ethanol. ZIF-8 was synthesised by reacting basic zinc carbonate (2.8 mmol) with 2-methylimidazole (64.4 mmol) in methanol, followed by centrifugation, washing, and drying at 120 °C overnight. UiO-66 was prepared from ZrCl_4_ (3.78 g), HCl (35%, 2.8 mL), and BDC (5.39 g) in DMF (98 mL) at 220 °C for 20 h, followed by DMF treatment at 100 °C for 150 min and drying at 60 °C overnight. MoS_2_ sheet material was synthesised based on our prior method, with an additional hydrothermal step [28,29]. Briefly, 500 mg of MoS_2_ was ball-milled with 10 g of KBr in a planetary ball mill (model PM200) using 3 mm zirconia balls at 500 rpm for 18 h, operated in cycles with 20 min milling intervals separated by 10 min rest periods. The resulting mixture was repeatedly washed with a DI water–ethanol solution (80:20 v/v) and dried in a vacuum oven at 60 °C to obtain the final powder. Three MOF-MoS_2_ hybrid materials were prepared using a shear-mixing method followed by sonication. The MOF-MoS_2_ ratio was optimised to 1:3 based on our previous work with graphene-based hybrids [26]. Typically, 150 mg of MoS_2_ and 50 mg of each MOF (CuBTC, ZIF-8 and UiO-66) in powder form were blended using a mortar and pestle for 20 min. The mixtures were then dispersed in ethanol, first subjected to mild bath sonication for 30 min, followed by tip sonication for an additional 30 min. These dispersions were subsequently used for sensor fabrication and material characterization.

### 2.3. Characterization of Materials

X-ray diffraction (XRD) measurements were performed using a Rigaku MiniFlex 600 X-ray diffractometer operating at a tube voltage of 40 kV and a tube current of 15 mA (Cu Kα radiation, λ = 1.5406 Å). JCPDS Card No. 00-062-1183 was used to identify the CuBTC crystal planes in the composite; Card No. 024-0513 was used to confirm the UiO-66-MoS_2_ composite; and JCPDS No. 00-062-1030 was used to identify the ZIF-8-MoS_2_ composite. Raman spectra of the MOF-MoS_2_ composite films were obtained using a HORIBA LabRam HR800 Evolution (Jobin Yvon, Horiba), with spectra recorded in the range of 500–4000 cm^−1^ under 532 nm laser excitation. Scanning electron microscopy (SEM) equipped with an energy-dispersive X-ray (EDAX) detector was conducted using a Quanta 450 microscope operated at an acceleration voltage of 10 kV and an emission current of 10 mA. Transmission electron microscopy (TEM) analysis was carried out using an FEI Titan Themis 80-200 microscope operated at 200 kV.

### 2.4. Fabrication and Characterisation of Sensors

The electrodes were fabricated by screen-printing Au paste onto a pre-patterned alumina substrate, followed by thermal annealing at 800 °C to form Au interdigitated electrodes (IDEs) comprising 11 electrode pairs (22 fingers). For sensor device fabrication, 3 wt.% of each MoS_2_-MOF hybrid powder, including CuBTC- MoS_2_, UiO-66-MoS_2_, and ZIF-8-MoS_2_, was dispersed in ethanol and sonicated in a bath for 30 min. The resulting suspensions were drop-coated (e.g., 100 μL) using a micropipette onto commercial interdigitated electrodes (IDEs). All coated samples were subsequently dried under vacuum at 80 °C to ensure reproducible and stable sensor performance. After fabrication, the sensors, with a sensing-film thickness of approximately 75–80 μm measured by optical profilometry, were stored at 30 °C under controlled environmental conditions prior to testing. The gas-sensing performance of the MOF–MoS_2_-based sensors, together with a MoS_2_-only control sensor, was evaluated by monitoring their chemoresistive responses under controlled flows of pure nitrogen (N_2_), ammonia (NH_3_), and volatile organic compound (VOC) vapours. VOC vapors were generated using a VOC generator (Owlstone V-OVG). Briefly, the liquid VOC was introduced into a diffusion tube placed inside the temperature-controlled oven of the V-OVG. A regulated flow of dry, high-purity N_2_ was passed over the diffusion tube to carry the generated VOC vapor. The VOC-containing gas stream was subsequently delivered to the sensing chamber through a flow-control system, allowing the desired VOC concentration to be precisely controlled during sensing measurements. Gas concentrations were precisely controlled using high-accuracy mass flow controllers, with the NH_3_ and VOC concentrations calculated and set according to Equation (1) below.

(1) $$\mathrm{Desired gas concentration}(\mathrm{ppm})=\frac{\left(\mathrm{Flow rate of gas}\right)\times \mathrm{Supplied gas concentration}(\mathrm{ppm})}{\left(\mathrm{Flow rate of gas}+\mathrm{Flow rate of nitrogen}\right)}$$

where the flow rate of gas or nitrogen is the volume of target gas or nitrogen passing into the sensing chamber per minutes (sccm). The total gas flow rate of target gas and nitrogen was maintained at 50 sccm throughout the measurements. The supplied gas concentration for VOC is fixed at 100 ppm, which is generated by the VOC generator using Equation (2).

(2) $$C_{\mathrm{VOC}}(\mathrm{ppm})=\frac{22.4\times \rho \times T\times V_{1}}{\mathrm{MW}\times 273\times V_{2}}\times 1000$$

where C_VOC_ is the VOC gas concentration (ppm), ρ is the density of VOC (g/mL), T is the operating temperature (K), MW is the molecular weight of the VOC (g/mol), V_1_ is the volume of the VOC solution (L), V_2_ is the volume of the chamber (L).

The target and interfering analytes included NH_3_, ethanol, methanol, acetone, and formaldehyde, with all measurements conducted at room temperature (25 °C). For NH_3_ sensing experiments, a certified gas cylinder containing 100 ppm NH_3_ in N_2_ (BOC Limited, North Ryde, NSW, Australia) was used. The NH_3_ concentration was adjusted by dilution with high-purity N_2_ using mass flow controllers (MFCs), allowing controlled delivery of the target gas to the sensing chamber. The total gas-flow rate was maintained at 50 sccm throughout the measurements.

Changes in device resistance upon exposure to each analyte were recorded using a Keithley 6517A electrometer connected to a computer for data acquisition and monitoring. The sensor response was defined according to Equation (3):

(3) $$A_{r}(\%)=\frac{R-R_{0}}{R_{0}}\times 100$$

where R_0_ is the initial resistance, and R is the resistance of the sensing materials after exposure to the analyzed vapours.

The sensors were subjected to eight exposure–recovery cycles, consisting of approximately 1 min exposure to NH_3_ or VOC vapour followed by 1 min purging with dry nitrogen, while the gas concentration was progressively varied from 100 ppm to 0.8 ppm. The corresponding changes in sensor resistance were recorded continuously. For the CuBTC–MoS_2_ hybrid, NH_3_ exposure produced an increase in resistance; therefore, the positive response values reported throughout this work represent the measured resistance change and were not obtained by absolute-value post-processing. To evaluate sensing stability and repeatability, the sensors were further tested over five consecutive cycles at 0.8 ppm. For each sensing material, at least three independent sensors were fabricated and tested to assess device-to-device reproducibility and variability in sensing performance.

For the humidity-interference experiments, high-purity nitrogen (N_2_) was used as the carrier gas. Water vapour was generated by passing N_2_ through a water bubbler, and the resulting humidified gas stream was mixed with dry N_2_ at controlled flow ratios to achieve the desired relative humidity (RH) levels. The sensing measurements were conducted at different RH levels, with each measurement repeated five times to evaluate reproducibility.

To determine the minimum detectable analyte concentration, the limit of detection (LOD) was calculated according to the IUPAC-recommended approach using the following Equation (4):

(4) $$\mathrm{LOD}=\frac{3\times \sigma_{\mathrm{baseline}}}{S}$$

where σ represents the standard deviation of the baseline signal obtained from ten replicate measurements (N = 10), and (S) is the slope of the linear calibration curve of sensor response versus analyte concentration.

### 2.5. Principal Component Analysis for Analyte Discrimination

Principal component analysis (PCA) was employed as an exploratory statistical method to analyse the gas/vapour sensing responses of three independently fabricated CuBTC–MoS_2_ devices exposed to five analytes. For each analyte, five repeated measurement cycles were performed on each device, resulting in 15 measurements per analyte and a total of 75 measurements included in the PCA dataset. Each measurement was represented as an individual data point based on its chemoresistive sensing response. PCA was used to reduce the dimensionality of the sensing data and examine differences in the response patterns toward the five tested gas/VOC analytes. The first two principal components (PC1 and PC2) together accounted for 90.37% of the total variance. The distribution of data points in the PCA plot revealed distinct response patterns among the tested analytes, indicating the potential for analyte discrimination. Nevertheless, PCA-based pattern recognition should be considered complementary to, rather than a replacement for, quantitative selectivity evaluation. Given the limited number of independently fabricated devices, these results should be regarded as exploratory, and further validation with a larger number of independent devices and measurements would be required to establish robust analyte classification.

## 3. Results

### 3.1. Characterisation of Hydrid Sensing Materials

**Figure 2:**
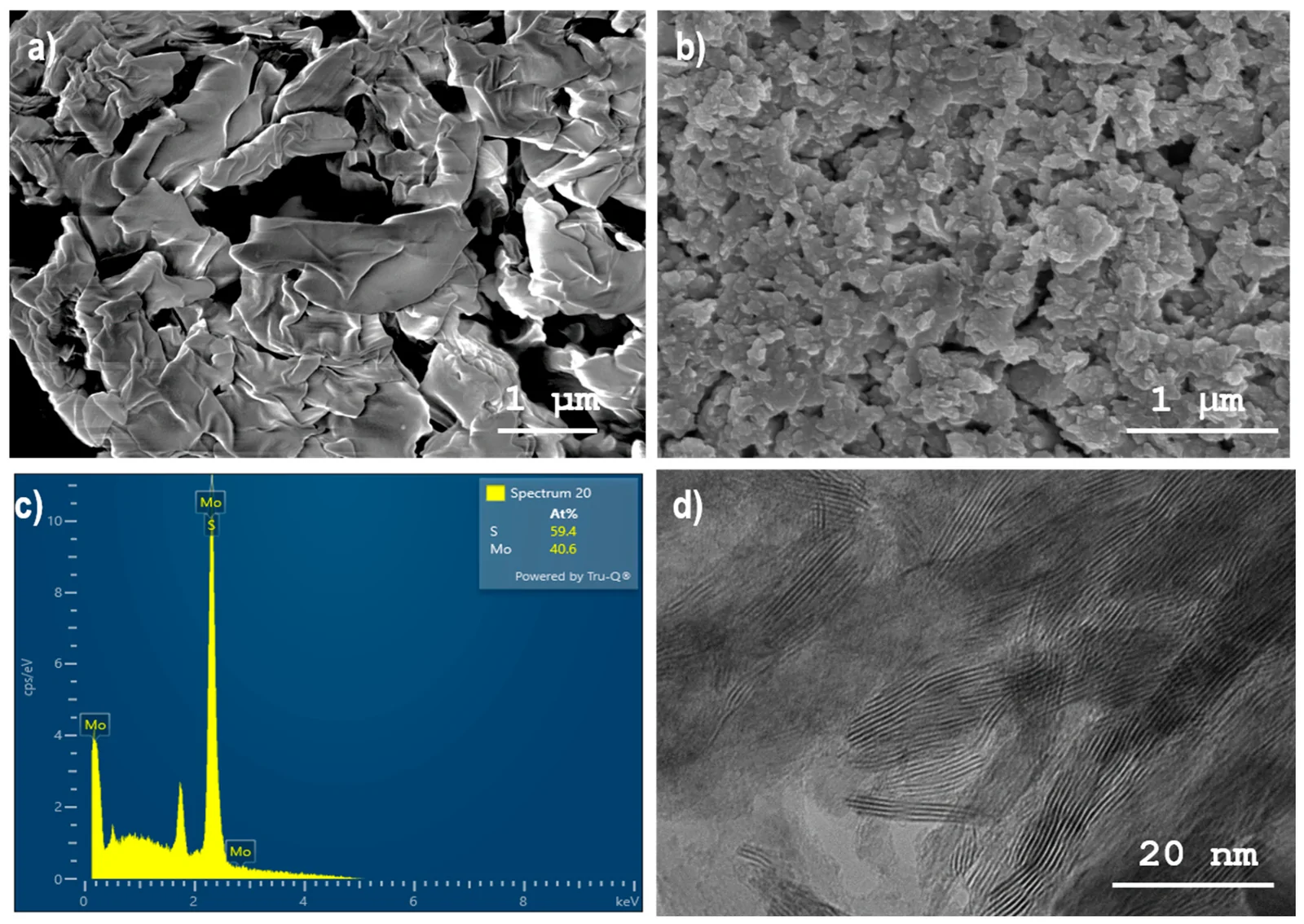
Micrograph of (**a**) bulk MoS_2_, (**b**) mechanically exfoliated MoS_2_ by ball milling, (**c**) typical EDX analysis showing the elements S and Mo in the exfoliated material, and (**d**) TEM image of few-layer MoS_2_, which presented 2–8 layers.

The morphological and structural characterisation of the prepared MoS_2_ materials is presented in Figure 2. The pristine bulk MoS_2_ precursor exhibits relatively thick, multilayered structures with particle sizes of approximately 2–4 μm (Figure 2a). Following mechanical milling, a substantial reduction in particle size (e.g., 0.5–1 μm) and layer thickness is observed, confirming effective exfoliation into thinner MoS_2_ nanosheets (Figure 2b). Meanwhile, the EDS spectrum (Figure 2c) confirms that the elemental composition of MoS_2_ is retained after exfoliation. The TEM image (Figure 2d) further reveals few-layer MoS_2_ nanosheets, typically consisting of approximately 2–8 layers. These characterisations confirm the successful delamination of bulk MoS_2_ into few-layer 2D nanosheets. The resulting reduction in lateral size and layer thickness is expected to increase the accessible surface area and active sites, providing a favourable platform for subsequent hybridisation with MOFs and enhanced gas-sensing performance.

**Figure 3:**
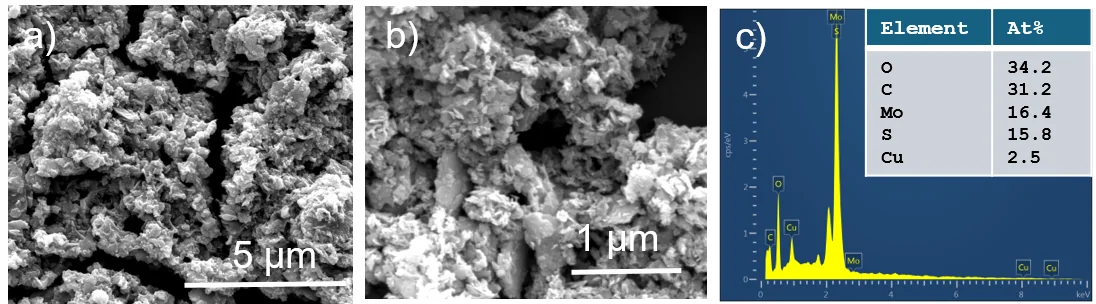
SEM images and EDX analysis of CuBTC-MoS_2_ hybrid material showing distribution of MoS_2_ sheets over the surface of CuBTC MOF.

The morphology, distribution, and elemental composition of the MOF-MoS_2_ hybrid materials are presented in Figure 3 and Figure S1 (Supporting Information (SI)). For CuBTC-MoS_2_, MoS_2_ nanosheets are randomly distributed across the CuBTC structures, attaching to well-defined, micrometer-sized CuBTC crystallites (Figure 3a,b). In contrast, UiO-66-MoS_2_, and ZIF-8-MoS_2_ (Figure S1, SI) show UiO-66 and ZIF-8 particles, respectively, randomly distributed over the MoS_2_ nanosheet surfaces. Mechanical shear mixing followed by ultrasonication reduces the MOF particles into smaller fragments, facilitating their attachment to and distribution across the MoS_2_ sheets through physical interactions. EDS analysis (Figure 3c) further confirms the characteristic elemental composition of each hybrid: Mo, S, O, Cu, and C for CuBTC-MoS_2_; Mo, S, O, Zr, and C for UiO-66-MoS_2_ (Figure S1c, SI), and Mo, S, Zn, N, O, and C for ZIF-8-MoS_2_ (Figure S1f, SI). These morphological and elemental analyses collectively confirm the successful formation of the three MOF-MoS_2_ hybrid materials.

**Figure 4:**
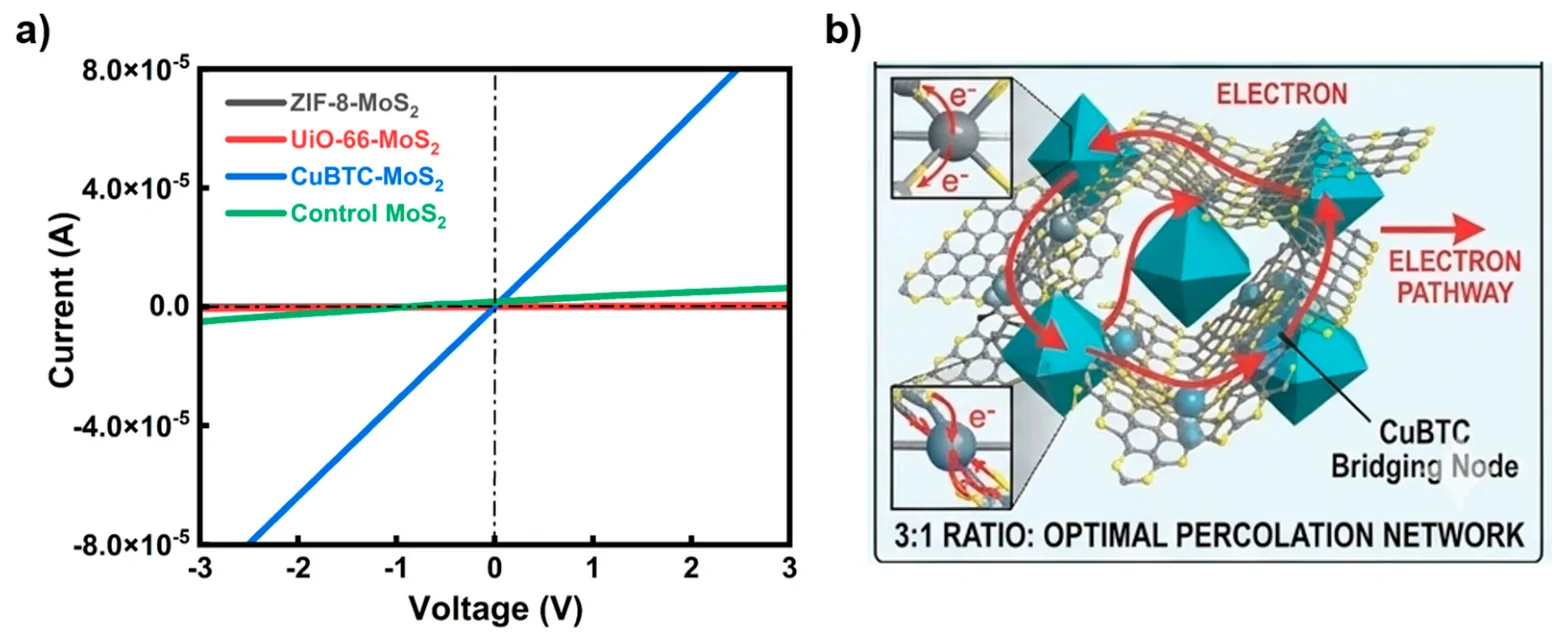
(**a**) IV curves of MOFs and MoS_2_ hybrid materials showing low conductivity of ZIF-8-MoS_2_, UiO-66-MoS_2,_ and control MoS_2_ only; linear Ohmic behavior of the optimized CuBTC-MoS_2_ hybrid material. (**b**) Schematic illustration of sonication- and bending-induced interfacial interactions enhancing conductivity in the optimised 1:3 CuBTC–MoS_2_ hybrid.

To evaluate the suitability of these materials for chemoresistive sensing, wherein the material’s resistance changes upon interaction with a target gas or vapour, the electrical conductivity of each hybrid was first examined. The I–V curves are shown in Figure 4. Exfoliated MoS_2_ alone exhibits low electrical conductivity, which becomes even poorer when hybridized with either UiO-66 or ZIF-8, as reflected in their respective I–V curves. Remarkably, when MoS_2_ is hybridized with CuBTC, a significant enhancement in electrical conductivity is observed, showing linear and symmetric current–voltage behaviour in the range of −5 to 5 V, indicative of well-defined Ohmic characteristics. This represents an excellent example of a “synergistic effect,” where two individually low-conductivity materials combine to yield a highly conductive sensing material. Room-temperature sensing tests toward ethanol vapor were performed on UiO-66-MoS_2_, ZIF-8-MoS_2_, and the MoS_2_ control. As expected from their low conductivity, UiO-66–MoS_2_ and ZIF-8–MoS_2_ exhibited no measurable sensing response, similar to pristine MoS_2_ (Figure S2, SI). In contrast, CuBTC–MoS_2_ showed significantly enhanced conductivity and was therefore selected for further gas-sensing characterisation. It is important to note that all three pristine MOFs: UiO-66, ZIF-8 are intrinsically non-conductive, and CuBTC is poorly conductive and they are therefore unsuitable as standalone chemoresistive sensing materials at room temperature. Remarkably, hybridisation of CuBTC with MoS_2_ produces a sufficiently conductive sensing network, highlighting the beneficial interfacial interactions between the two components. Accordingly, subsequent sensing investigations focus exclusively on the CuBTC–MoS_2_ hybrid. Herein, the superior conductivity of the CuBTC-MoS_2_ hybrid, compared to ZIF-8- and UiO-66-based systems, arises from the distinct electronic nature of the CuBTC framework. ZIF-8 (Zn^2+^) and UiO-66 (Zr^4+^) are largely redox-inactive, possess fully coordinated metal nodes, and exhibit intrinsically insulating behavior (wide band gaps), limiting their ability to participate in charge transport. The enhanced electrical conductivity of the CuBTC–MoS_2_ hybrid, despite the intrinsically insulating nature of pristine CuBTC, can be attributed to synergistic structural and interfacial effects that improve charge transport through the semiconducting MoS_2_ network [30], as hypothesized in the mechanism in Figure 4b. At the 3:1 MoS_2_ ratio, MoS_2_ forms a continuous conductive matrix around the dispersed CuBTC crystals, while the rigid MOF particles act as spacers that suppress MoS_2_ restacking and increase the exposure of conductive edges and interfacial sites. Intimate contact between the two components further promotes interfacial charge transfer, where redox-active Cu^2+^/Cu^+^ centers and coordinatively unsaturated Cu sites can mediate charge exchange with MoS_2_ [31,32,33]. Possible coordination between S atoms of MoS_2_ and open Cu sites may additionally strengthen electronic coupling across the heterointerface and reduce charge-transfer resistance (Figure S3, SI). Interfacial charge redistribution and Fermi-level equilibration may also modify the carrier concentration and transport barriers within MoS_2_ [34]. Furthermore, structural distortion and strain induced by the rigid CuBTC particles may locally modify the electronic structure of MoS_2_ and potentially promote defect formation or localized 2H-to-1T phase transformation, providing additional conductive pathways. Collectively, the improved percolation network, suppressed restacking, interfacial charge transfer, and local electronic modulation provide a plausible explanation for the enhanced conductivity of the CuBTC–MoS_2_ hybrid.

**Figure 5:**
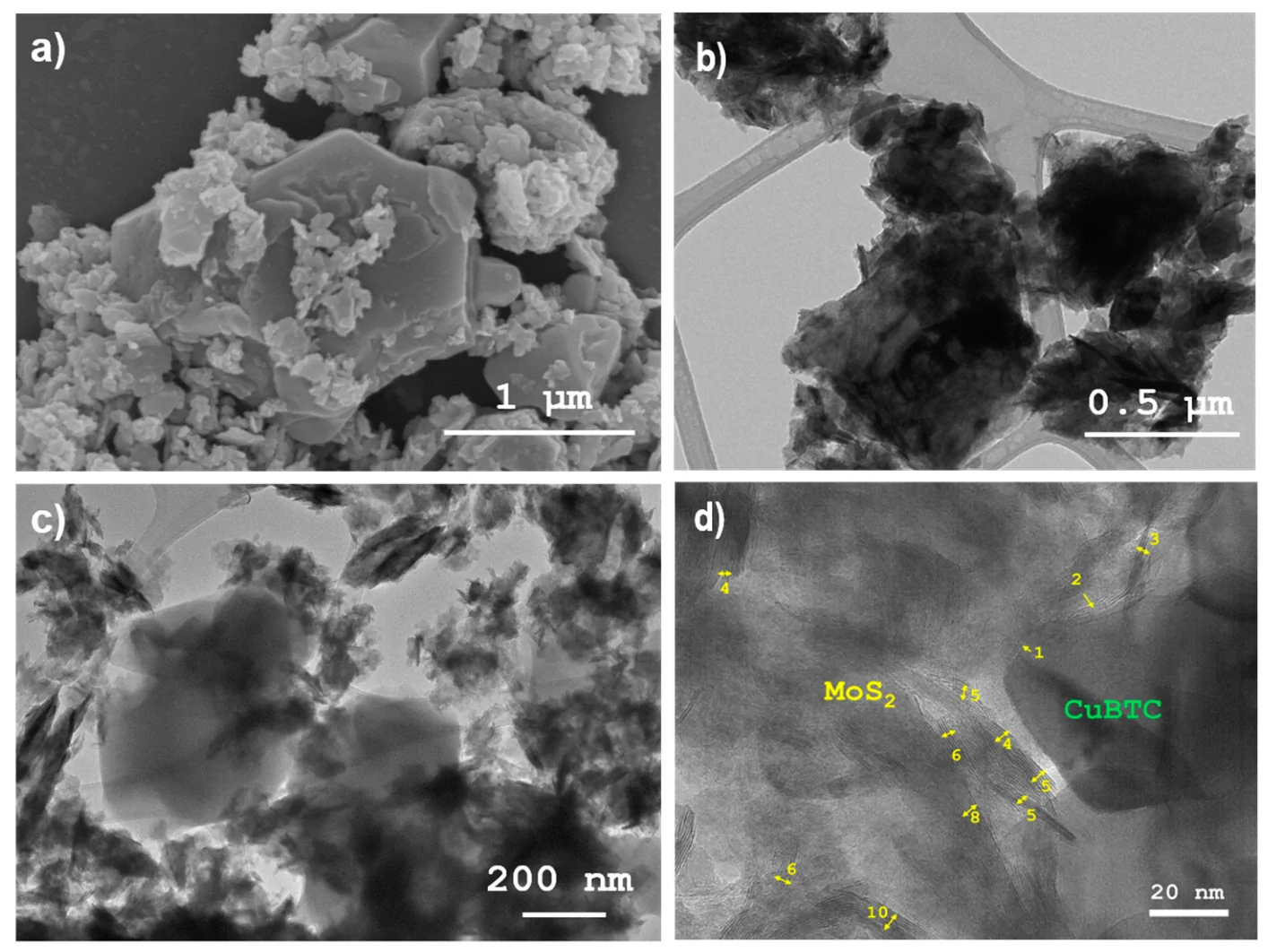
(**a**) SEM image of mechanically exfoliated MoS_2_, (**b**) corresponding TEM image showing (**c**) MoS_2_-coated CuBTC and (**d**) the random immobilisation of MoS_2_ nanosheets on CuBTC crystals, (**d**) the few-layered structure of 2D MoS_2_ nanosheets and CuBTC crystals.

Figure 5 presents magnified SEM images (Figure 5a) and TEM images (Figure 5 b–d) of the CuBTC–MoS_2_ hybrid. The SEM image shows that mechanical milling followed by ultrasonication effectively delaminates bulk, several-micron-sized MoS_2_ into smaller (e.g., hundreds of nm), uniformly distributed nanoscale flakes. TEM analysis further confirms the formation of few-layer MoS_2_ nanosheets, which are observed both coating the CuBTC surface (Figure 5b) and randomly immobilised on well-defined CuBTC crystals (Figure 5c,d), forming a heterogeneous hybrid structure. This intimate and randomly distributed contact between MoS_2_ and CuBTC indicates effective hybridisation, providing abundant interfacial regions and accessible active sites that are expected to promote molecular adsorption and charge-transfer interactions for enhanced gas sensing.

**Figure 6:**
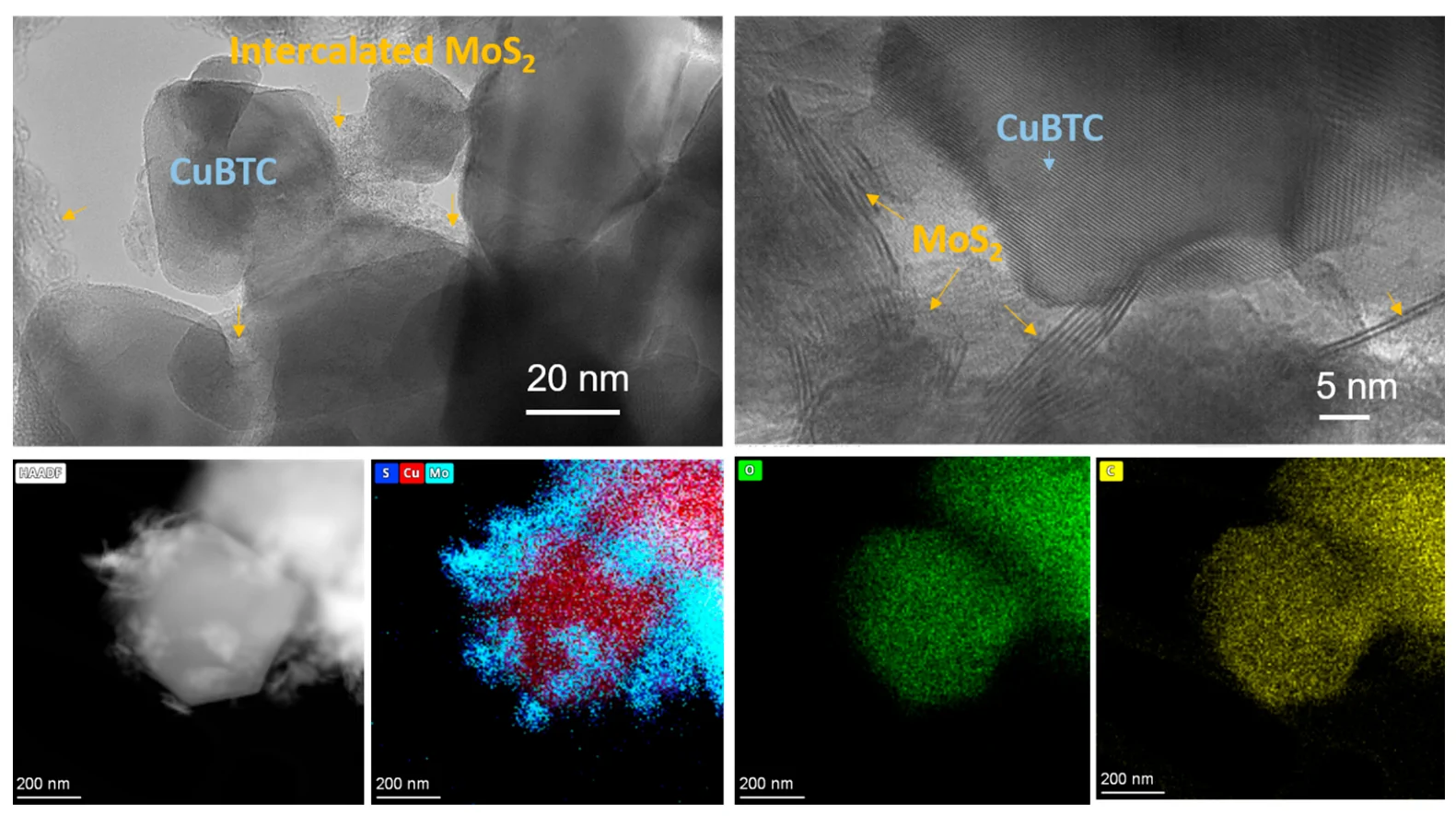
TEM of CuBTC-MoS_2_ hybrid materials, which showed distribution of MoS_2_ sheets, and CuBTC particles, wherein the sheets intercalated between CuBTC particles and covered on their surface. The HAADF and EDX element analysis presented the hybridization as well.

The morphology and heterostructure of the CuBTC-MoS_2_ hybrid were further examined with RH-TEM analysis (Figure 6, top). MoS_2_ flakes (10–50 nm) are uniformly incorporated within CuBTC particles (50–250 nm), forming an interconnected network. Single- to few-layer MoS_2_ flakes attach to CuBTC crystallites, resulting in a hierarchical porous structure. The HR-TEM images clearly reveal the interface between CuBTC and MoS_2_, which is critical for charge transfer in sensing applications. High-angle annular dark-field scanning transmission electron microscopy (HAADF-STEM, Figure 6, bottom) was performed to obtain atomic-scale compositional information. Brighter contrast indicates regions with higher atomic numbers, enabling the visualization of Mo and S in the MoS_2_ sheets and Cu in CuBTC. Elemental mapping confirms the uniform distribution of Mo, S, and Cu across the hybrid, validating the formation of the CuBTC-MoS_2_ heterostructure. HAADF-STEM also reveals how CuBTC particles influence the morphology and edges of MoS_2_ nanosheets, highlighting the intimate interface between the two components. This interface plays a key role in facilitating electronic interactions and enhancing charge transport.

Overall, TEM and HAADF-STEM analyses demonstrate that CuBTC crystals and MoS_2_ nanosheets form a well-integrated heterostructure. This architecture contributes to the modified electronic structure and underpins the significantly improved electrical conductivity of the CuBTC-MoS_2_ hybrid.

**Figure 7:**
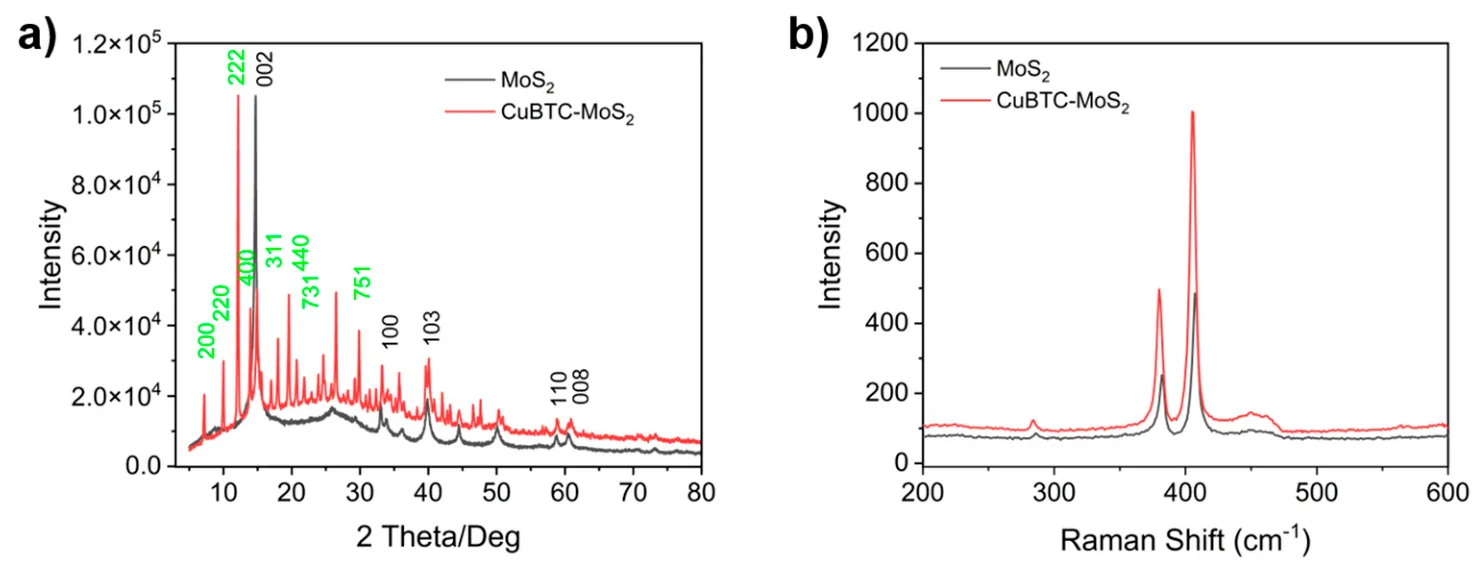
(**a**) XRD and (**b**) Raman of control MoS_2_ and CuBTC-MoS_2_ hybrid materials.

The X-ray diffraction (XRD) pattern of the CuBTC-MoS_2_ hybrid shows a combination of diffraction peaks from both the CuBTC framework and MoS_2_ (Figure 7a). The sharpness and intensity of the peaks indicate the high crystallinity of the hybrid, while their positions confirm the presence of the expected crystal phases. Characteristic peaks of CuBTC, corresponding to the (200), (220), (222), (311), (440), (731), and (751) planes, are observed alongside the MoS_2_ peaks from its hexagonal structure, including (002), (100), (103), (110), and (008) [19]. In the hybrid, the XRD pattern appears as a superposition of both materials, with slight peak shifts or broadening likely resulting from interactions between CuBTC and MoS_2_.

Raman spectra further support the hybrid formation (Figure 7b). The CuBTC-MoS_2_ hybrid exhibits the characteristic MoS_2_ peaks, with the E_2g_^1^ mode observed at ~380 cm^−1^ and the A_1g_ mode at ~406 cm^−1^, showing slight shifts compared to pristine MoS_2_ (E_2g_^1^~382 cm^−1^, A_1g_ ~408 cm^−1^) [35]. Peaks corresponding to CuBTC, such as carbon–carbon vibrations, are not distinctly observed in the hybrid. The observed peak shifts indicate strong interactions between CuBTC and MoS_2_, confirming the formation of a well-integrated hybrid structure.

### 3.2. Evaluation of Chemoresistive Sensing Performance

**Figure 8:**
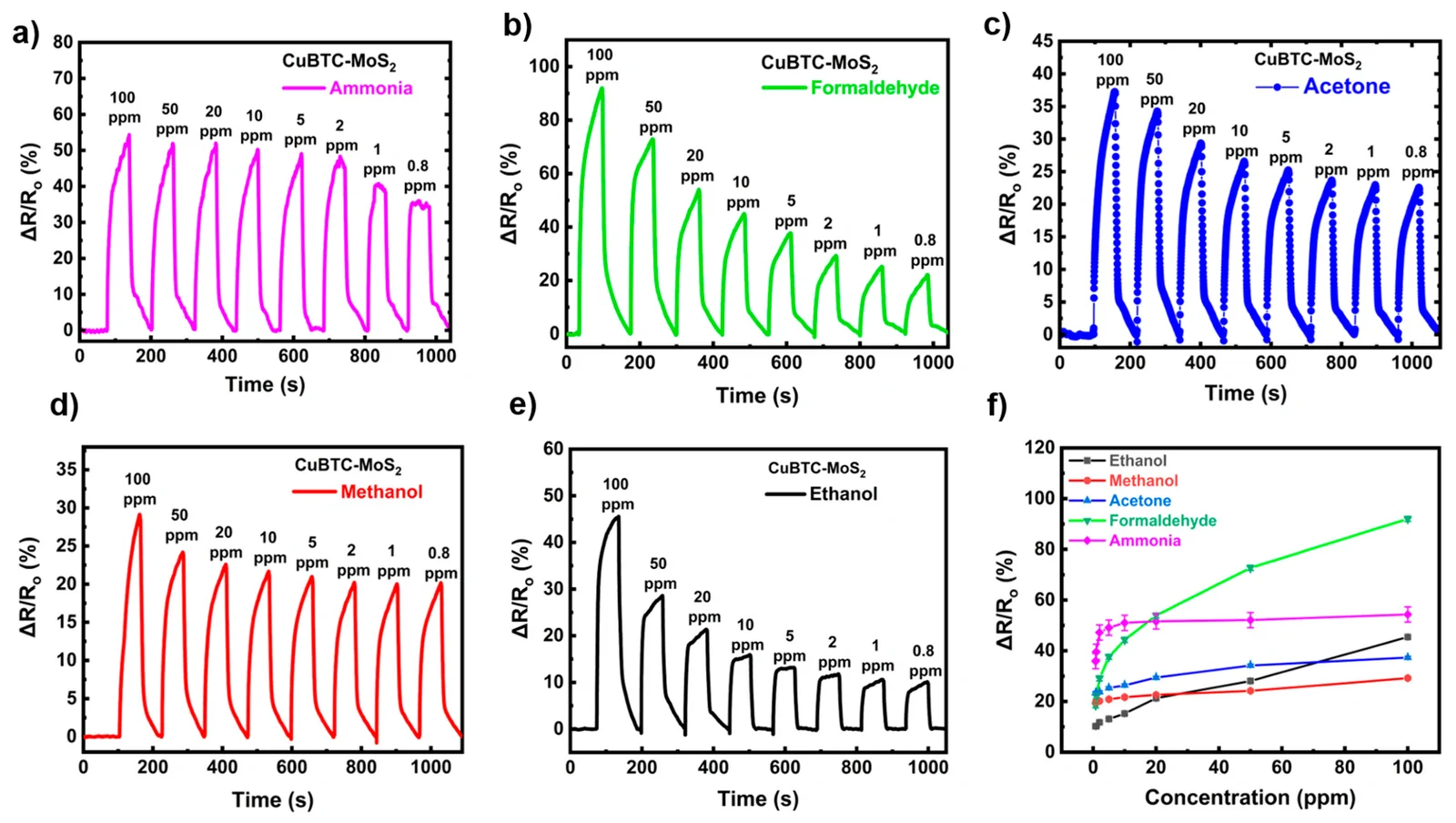
Room-temperature sensing responses of the CuBTC–MoS_2_ hybrid sensor toward (**a**) ethanol, (**b**) methanol, (**c**) acetone, (**d**) formaldehyde, and (**e**) ammonia over concentrations ranging from 100 ppm to 0.8 ppm; (**f**) comparison of the sensing responses toward all tested analytes.

The CuBTC–MoS_2_ hybrid was evaluated for room-temperature chemoresistive sensing of ammonia (NH_3_) and selected volatile organic compounds (VOCs), including ethanol, methanol, acetone, and formaldehyde, over concentrations ranging from 0.8 to 100 ppm, as shown in schematic of testing setup (Figure S4, SI). The sensor exhibited measurable and concentration-dependent responses toward all tested analytes, demonstrating the broad gas-sensing capability of the CuBTC–MoS_2_ hybrid (Figure 8). As shown in Figure 8a–c, the responses toward ethanol, methanol, and acetone reached 45.5%, 29.2%, and 37.7%, respectively, at 100 ppm and generally decreased with decreasing analyte concentration toward the sub-ppm range. Differences in response among these VOCs may be associated with their distinct molecular properties and adsorption interactions with the CuBTC–MoS_2_ surface, which influence adsorption-induced charge transfer and the resulting resistance modulation. Below approximately 35 ppm, the response toward ethanol became lower than that toward methanol, suggesting concentration-dependent differences in adsorption and surface interactions. Such behaviour may also be influenced by competitive adsorption at the available active sites, particularly at low analyte concentrations.

Notably, the CuBTC–MoS_2_ sensor exhibited pronounced responses toward both formaldehyde and NH_3_, reaching 92.3% and 54.6% at 100 ppm, respectively (Figure 8d,e). The concentration-dependent responses exhibited nonlinear behaviour, particularly in the low-concentration range (0.8–10 ppm), while the NH_3_ response increased slightly at higher concentrations (10–100 ppm), suggesting an approach toward saturation (Figure 8f). Interestingly, the sensor exhibited a higher response toward NH_3_ than formaldehyde at concentrations below approximately 17 ppm, whereas formaldehyde produced a stronger response at higher concentrations. This concentration-dependent crossover indicates that CuBTC–MoS_2_ is not exclusively selective toward NH_3_ but exhibits broader gas-sensing capabilities. The pronounced formaldehyde response may be attributed to favourable interactions with accessible active sites within the hybrid, facilitating adsorption-induced charge transfer and resistance modulation. Meanwhile, NH_3_ sensing is likely associated with interactions involving coordinatively unsaturated Cu sites in CuBTC, coupled with interfacial charge transfer and carrier modulation in MoS_2_. These findings highlight the important roles of analyte identity and concentration in determining the sensing behaviour of CuBTC–MoS_2_. It should also be noted that, although the 60 s exposure duration captured the major resistance changes under the investigated conditions, complete response saturation was not necessarily achieved, particularly at higher analyte concentrations, as shown in Figure S5 (SI). Therefore, the reported responses represent resistance changes measured after a fixed exposure duration rather than fully equilibrated steady-state responses.

Despite its response toward selected VOCs, the pronounced NH_3_ response extending into the sub-ppm range remains particularly relevant to ammonia leakage monitoring in hydrogen-carrier applications. Rapid and sensitive detection at low NH_3_ concentrations is important for improving the safety of ammonia storage, transportation, and utilisation. Therefore, while the present results demonstrate cross-sensitivity toward selected VOCs, they also highlight the potential of CuBTC–MoS_2_ as a room-temperature NH_3_ sensing platform for ammonia-based hydrogen-carrier safety, with further optimisation of analyte discrimination required for operation in complex gas environments.

**Figure 9:**
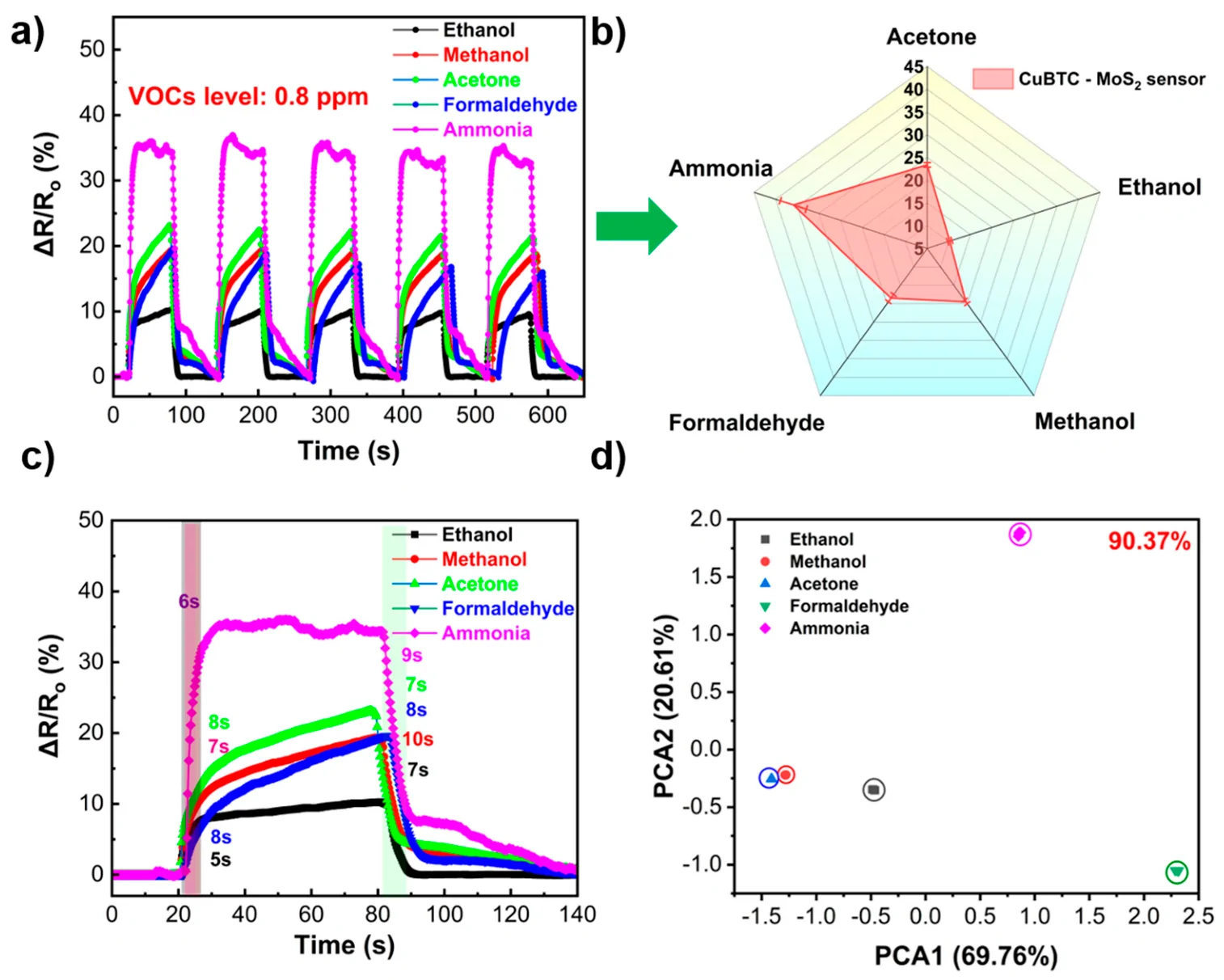
(**a**) Sensing response of CuBTC-MoS_2_ sensor at room temperature towards different VOCs at 0.8 ppm concentration. (**b**) Selectivity of the sensor at low concentrations of sub-ppm VOCs. (**c**) Response time and recovery time of the sensor at 0.8 ppm VOC level. (**d**) PCA score plot illustrating the discrimination of the investigated analytes based on their sensing responses. Five repeated measurement cycles were performed for each analyte on each sensor, yielding 15 measurements per analyte. Each data point represents an individual sensing measurement.

At the lowest tested concentration (0.8 ppm), the CuBTC–MoS_2_ sensor exhibits the highest response toward ammonia (~35%), followed by acetone (~23.5%), methanol (~19.5%), formaldehyde (~18.5%), and ethanol (~10.2%) (Figure 9a). The pronounced response toward NH_3_ is likely associated with its strong electron-donating character, arising from the lone electron pair on the nitrogen atom, which facilitates adsorption-induced charge transfer with the CuBTC–MoS_2_ sensing material. In contrast, the other analytes exhibit different adsorption affinities and charge-transfer interactions with the hybrid surface, resulting in comparatively lower responses. Importantly, the preferential response toward sub-ppm NH_3_ highlights the potential of CuBTC–MoS_2_ for sensitive room-temperature ammonia leakage monitoring in hydrogen-carrier storage, transportation, and utilisation systems.

The highest NH_3_ response at sub-ppm level is attributed to the synergistic effects of selective adsorption, charge transfer, and carrier modulation within the CuBTC–MoS_2_ hybrid. NH_3_ molecules preferentially interact with coordinatively unsaturated Cu sites in CuBTC through Lewis acid–base interactions, effectively concentrating NH_3_ near the CuBTC–MoS_2_ interface. Upon adsorption, NH_3_ acts as an electron donor, increasing the carrier concentration in n-type MoS_2_ and consequently decreasing its resistance. The intimate interfacial contact between CuBTC and MoS_2_ facilitates efficient transfer of adsorption-induced charge into the conductive MoS_2_ network, thereby amplifying the chemoresistive response. Meanwhile, the highly porous structure and large surface area of CuBTC promote gas diffusion and provide abundant accessible adsorption sites, contributing to the high sensitivity and rapid response toward NH_3_, particularly at low concentrations.

The observed selectivity toward ammonia is further governed by the combined structural and electronic characteristics of the hybrid. The porous CuBTC framework promotes preferential adsorption of NH_3_ due to its molecular size, polarity, and strong affinity for Cu open metal sites, while MoS_2_ serves as an efficient transducer that converts adsorption-induced charge variations into measurable electrical signals. In contrast, the interfering VOCs exhibit weaker adsorption and/or less effective charge-transfer interactions, resulting in lower sensing responses. Collectively, the preferential adsorption of NH_3_ by CuBTC and efficient electrical transduction through MoS_2_ account for the enhanced sensitivity and selectivity of the hybrid sensor toward ammonia.

The selectivity of the CuBTC-MoS_2_ sensor toward the tested VOCs at 0.8 ppm is plotted in Figure 9b, showing a strong and distinct response to ammonia compared to other VOCs. This excellent selectivity toward ammonia (at sub-ppm level) is a key characteristic of the CuBTC-MoS_2_ hybrid material. The specific selectivity profile, however, can vary depending on factors such as the sensing material, VOC concentration, sensor design and fabrication, and operating conditions.

The response and recovery characteristics of the CuBTC–MoS_2_ sensor toward 0.8 ppm NH_3_ are presented in Figure 9c. The response time (t_90_) is defined as the time required for the sensor signal to reach 90% of the maximum resistance change recorded during NH_3_ exposure, whereas the recovery time is defined as the time required for the signal to return to 10% of this maximum resistance change following NH_3_ removal, corresponding to 90% recovery toward the baseline. Under the experimental conditions employed, including a fixed NH_3_ exposure duration of 60 s, the response and recovery times were approximately 6 s and 9 s, respectively. It should be emphasised that these values are determined relative to the maximum response recorded within the specified exposure period and may vary with exposure duration, particularly when the sensor has not reached a fully equilibrated response.

The sensor exhibits a fast response of 5–8 s and a recovery time of 7–10 s. This is notably rapid, especially compared to sensors based solely on MOFs or MoS_2_. For example, MoS_2_ composite-based sensors show response and recovery times of 400 s and 280 s, respectively, for ammonia, while another MoS_2_-based composite sensor exhibited response and recovery times of 65 s and 70 s [36]. A MoS_2_-CuO nanocomposite demonstrated a response time of 61 s and a recovery time of 85 s for 10 ppm acetone at room temperature [36]. A V_2_CTₓ-MoS_2_ composite sensor showed a recovery time exceeding 30 s for low concentrations of ammonia at room temperature [37]. Furthermore, a MoS_2_ VOC diode sensor exhibited a response time of approximately 10 s to 200 ppm acetone under UV light at room temperature [38].

Overall, the fast response and recovery times of the CuBTC-MoS_2_ hybrid are among the highest reported for MoS_2_-based sensors and are significantly superior to CuBTC-only sensors, which generally exhibit response and recovery times on the order of tens to hundreds of seconds [22,39,40].

The signal-to-noise ratio (SNR) is an important parameter for evaluating the reliability and quality of sensor signals, particularly at low analyte concentrations. In this study, the SNR was calculated as the ratio of the sensor response to the standard deviation of the baseline noise (as indicated SI). The calculated SNR values of the CuBTC–MoS_2_ sensor toward different NH_3_ concentrations are summarized in Table S1 (SI). The SNR increased progressively from 784.30 at 0.8 ppm to 1289.23 at 100 ppm, indicating a concentration-dependent increase in the sensor signal relative to the baseline noise. Notably, even at the lowest investigated NH_3_ concentration of 0.8 ppm, the sensor exhibited a remarkably high SNR of 784.30, demonstrating a clearly distinguishable sensing response against the baseline fluctuations.

This study demonstrates that the hybrid material-based sensor exhibits distinct response magnitudes toward different VOCs, with particularly strong responses toward ammonia at trace levels (less than 10 ppm). The ratio and morphology of the CuBTC and MoS_2_ components play a crucial role in determining the final selectivity. The high response to ammonia is primarily attributed to its chemical properties: ammonia is more polarized than many other gases due to its lone electron pair, which promotes stronger interactions with the sensing material.

**Figure 10:**
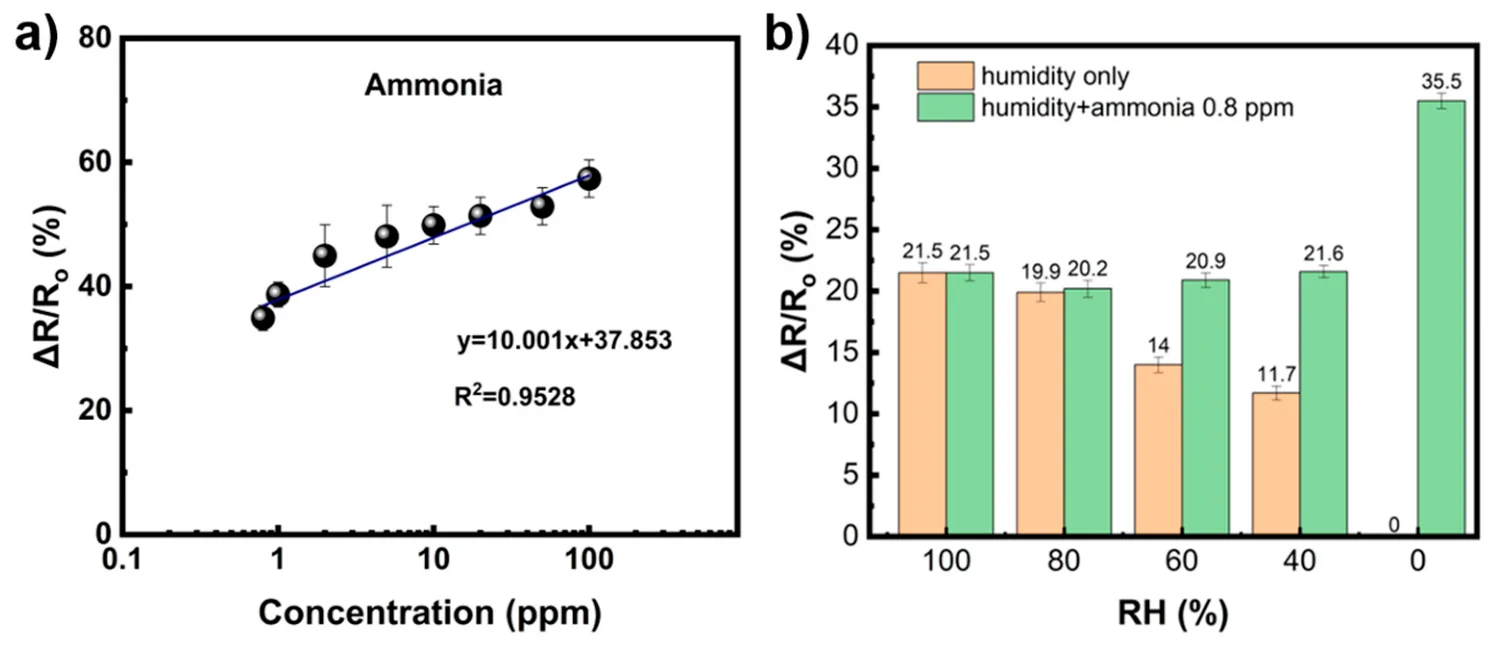
(**a**) Calculated limit of detection for ammonia; (**b**) Effect of relative humidity (RH) on the CuBTC–MoS_2_ sensor response under different RH conditions, both in the absence and presence of 0.8 ppm NH_3_ at room temperature.

The PCA map (Figure 9d) represents the multidimensional sensor responses to different VOCs projected onto a reduced-dimensional space (typically PC1 and PC2), enabling clear visualization of pattern separation. For the CuBTC–MoS_2_ sensor, the well-separated clusters indicate good selectivity and distinct response signatures for most tested VOCs, while the close proximity of the methanol and acetone clusters suggests limited discrimination between these two analytes. This is likely because both act as reducing gases with high polarity and hydrogen-bonding affinity. They therefore exhibit similar adsorption behavior and charge-transfer interactions, as well as comparable diffusion and mass-transport kinetics across the porous sensing material. In short, the PCA results demonstrate distinct response patterns among the target gases, with minor overlap for chemically similar species, thereby complementing the quantitative selectivity evaluation. Overall, the high selectivity toward NH_3_ makes the CuBTC–MoS_2_ sensor particularly promising for reliable ammonia leakage monitoring in hydrogen-carrier storage, transportation, and utilisation systems, while minimising interference from other gases and VOCs. The limit of detection (LOD) is defined as the lowest analyte concentration that can be reliably distinguished from the baseline noise [41]. In this study, the limit of detection (LOD) was estimated using Equation (4), and extrapolation from the linear calibration curve (Figure 10a). The calculated LOD for NH_3_ was 0.013 ppm (13 ppb), highlighting the high sensitivity of the CuBTC–MoS_2_ sensor and its potential for trace-level ammonia detection at room temperature. This low detection limit highlights the promising applicability of the developed sensing material for environmental monitoring and other applications requiring ultralow-concentration gas detection. Figure 10b presents the responses of the CuBTC–MoS_2_ sensor at different relative humidity (RH) levels, in the absence and presence of 0.8 ppm NH_3_ at room temperature, with the corresponding transient response profiles provided in Figure S6 (SI). Without NH_3_, the sensor exhibits a humidity-dependent response, increasing from 11.7% to 21.5% as RH rises from 40% to 100%, with a smaller increase between 80% and 100% RH, indicating significant sensitivity to moisture. In the presence of 0.8 ppm NH_3_, the transient response decreases markedly from 35.5% under dry N_2_ conditions (0% RH) to 21.6% at 40% RH, followed by only a slight further reduction up to 100% RH. This behaviour suggests that water adsorption influences the sensing response, potentially through competition with NH_3_ for accessible adsorption sites and modification of interfacial charge-transfer processes, with the humidity-induced suppression becoming less pronounced at higher RH levels.

Humidity interference could be mitigated through surface functionalisation or hydrophobic modification of the CuBTC–MoS_2_ hybrid to reduce water adsorption while maintaining NH_3_ accessibility. Humidity compensation using a reference humidity sensor or signal-correction algorithm could further improve sensing accuracy under variable environmental conditions [42]. Importantly, prolonged exposure to high humidity may also compromise the hydrolytic stability of CuBTC because water molecules can interact with the Cu coordination sites and progressively disrupt the Cu–carboxylate framework. Therefore, long-term stability under cyclic and sustained high-humidity conditions requires further investigation for practical deployment. Protective hydrophobic coatings or surface modification may provide potential strategies for simultaneously improving hydrolytic stability and reducing humidity interference. Operation at moderately elevated temperatures may also facilitate water desorption; however, this approach should be carefully optimised to preserve the advantages of low-power, near-room-temperature NH_3_ sensing.

To examine the stability of the CuBTC–MoS_2_ sensor, its sensing performance toward 0.8 ppm NH_3_ was monitored over a period of two weeks, as shown in Figure S7 (SI). A minor decrease of 5.3% in the response magnitude was observed over the testing period. This limited degradation demonstrates the good stability of the composite sensing material under ambient conditions, which is important for reliable gas-sensing applications.

## 4. Conclusions

In this study, three metal–organic frameworks (CuBTC, ZIF-8, and UiO-66) were hybridised with MoS_2_ at an optimised MOF:MoS_2_ ratio of 1:3 (w/w) and evaluated as room-temperature chemoresistive chemical vapour sensors. While ZIF-8–MoS_2_ and UiO-66–MoS_2_ exhibited poor electrical conductivity and limited sensing capability, CuBTC–MoS_2_ demonstrated a pronounced and reproducible sensing response, highlighting the critical role of MOF chemistry and interfacial interactions in determining the sensing performance of MOF–MoS_2_ hybrids. CuBTC–MoS_2_ exhibited concentration-dependent responses toward NH_3_ and selected VOCs, including ethanol, methanol, acetone, and formaldehyde, over the concentration range of 0.8–100 ppm. Among the VOCs, the sensor exhibited the highest transient response to formaldehyde at concentrations above 17 ppm, indicating broader gas-sensing capability. Meanwhile, the sensor exhibited the strongest response to NH_3_ at concentrations below approximately 17 ppm, particularly in the trace-concentration range of 0.8–10 ppm, demonstrating the preferential sensitivity of CuBTC–MoS_2_ toward trace concentrations of NH_3_. Notably, the CuBTC–MoS_2_ sensor exhibited a high response of approximately 35% toward 0.8 ppm NH_3_, accompanied by an excellent signal-to-noise ratio, an ultralow calculated limit of detection (LOD) of 0.013 ppm (13 ppb), and rapid response and recovery times of 6 s and 9 s, respectively, demonstrating its promising potential for highly sensitive and reliable NH_3_ detection at room temperature. The enhanced NH_3_ response is attributed to favourable adsorption at coordinatively unsaturated Cu sites, adsorption-induced charge transfer, and carrier modulation within MoS_2_, while the porous CuBTC structure facilitates molecular diffusion and access to active sites.

Overall, these results demonstrate the effectiveness of integrating the molecular adsorption capabilities of CuBTC with the electrical transduction properties of 2D MoS_2_ for room-temperature gas sensing. The enhanced sensing performance of the CuBTC–MoS_2_ hybrid can be attributed to the combined effects of reduced MoS_2_ restacking, improved charge-transport pathways, and interfacial charge-transfer interactions. Importantly, the hybrid exhibits a preferential response toward NH_3_ over the other investigated analytes in the trace-concentration range (0.8–10 ppm), highlighting its potential for sensitive and rapid NH_3_ detection at room temperature. Its pronounced sub-ppm NH_3_ response, together with rapid response and recovery kinetics, makes CuBTC–MoS_2_ a promising sensing material for the early detection of ammonia leakage, particularly in applications involving NH_3_ storage, transportation, and utilisation as a hydrogen carrier. Further investigations under mixed-gas conditions and varying environmental conditions will be valuable for assessing its practical applicability in complex sensing environments.

## Data Availability

The original contributions presented in this study are included in the article/Supplementary Materials. Further inquiries can be directed to the corresponding authors.

## Supplementary Materials

The following supporting information can be downloaded at: https://www.mdpi.com/article/10.3390/s26217082/s1, Figure S1: SEM images and EDX analysis of (a–c) UiO-66-MoS_2_, and (d-f) ZIF-8-MoS_2_. Figure S2: Chemoresistive sensing test of (top-left) ZIF-8-MoS_2_, (top-right) UiO-66-MoS_2_ and (bottom) control MoS_2_ only materials, showing no response to ammonia at room temperature. Figure S3: Schematic illustration of the proposed interaction mechanism within the optimized 1:3 CuBTC-MoS­_2_ architecture under mechanical bending-induced strain, in which led to possible coordination between S atoms of MoS₂ and open Cu sites, and transitioned 2H to 1t phase at the interphases. Figure S4: A schematic diagram of the experimental setup for VOCs tests. Figure S5: Sensing tests using exposure periods of 1, 3, 5 and 8 min a similar overall transient-response profile, with only a relatively small additional resistance change during prolonged exposure. Figure S6: Effect of relative humidity (RH) on the CuBTC–MoS₂ sensor at room temperature: (a) sensing responses under different RH levels in the absence of NH₃; and (b) sensing responses toward 0.8 ppm NH₃ under varying RH conditions. Figure S7: Stability test of CuBTC-MoS2 sensor to ammonia chemical vapor at 0.8 ppm for a prior of two weeks. Table S1: Calculation of signal-to-noise ratio (SNR) versus tested ammonia gas concentration.
