# Supplementary material for: Hybrid 2D MoS2–Copper Benzene-1,3,5-Tricarboxylate Framework for Room-Temperature Gas Sensing: Toward Trace Ammonia Detection for Hydrogen-Carrier Safety
Source: Sensors (Basel). 2026 Nov 9;26(21):7082. doi: 10.3390/s26217082 (PMC13766472; doi:10.3390/s26217082)
Supplement: Supplementary file 1 [file sensors-26-07082-s001.zip › sensors-4546560-supplementary.pdf]

sensors

*Supplementary Materials*

# Hybrid 2D $MoS_2$–Copper Benzene-1,3,5-tricarboxylate Framework for Room-Temperature Gas Sensing: Toward Trace Ammonia Detection for Hydrogen-Carrier Safety

**Tran Thanh Tung [1,*], Anh Tuan Trong Tran [1], Md Abdul Jalil [1], Tran Van Khai [2,3], Thuy Ai Huyen Le [4], Duc Thuan Lao [4], Mickael Castro [5], Jean-Francois Feller [5], Nguyen Xuan Sang [6,7] and Dusan Losic [1,*]**

1 School of Chemical Engineering, Adelaide University, Adelaide, SA 5005, Australia; trongtuananh.tran@adelaide.edu.au (A.T.T.T.); mdabdul.jalil@adelaide.edu.au (M.A.J.)
2 VNU-HCM Key Laboratory for Material Technologies, Ho Chi Minh City University of Technology (HCMUT), 268 Ly Thuong Kiet Street, Dien Hong Ward, Ho Chi Minh City 70000, Vietnam; tvkhai1509@hcmut.edu.vn
3 Vietnam National University—Ho Chi Minh City (VNU-HCM), Quater 33, Linh Xuan Ward, Ho Chi Minh City 71308, Vietnam
4 Center for Life Science Research, Ho Chi Minh City Open University, 97 Vo Van Tan, Xuan Hoa Ward, Ho Chi Minh City 70000, Vietnam; thuy.lha@ou.edu.vn (T.A.H.L.); thuan.ld@ou.edu.vn (D.T.L.)
5 Smart Plastics Group, IRDL CNRS 6027, University of South Brittany (UBS), 56321 Lorient, France; mickael.castro@univ-ubs.fr (M.C.); jean-francois.feller@univ-ubs.fr (J.-F.F.)
6 Atomic Molecular and Optical Physics Research Group, Institute for Advanced Study in Technology, Ton Duc Thang University, Ho Chi Minh City 72912, Vietnam; nguyen-xuansang@tdtu.edu.vn
7 Faculty of Electrical and Electronics Engineering, Ton Duc Thang University, Ho Chi Minh City 70000, Vietnam
* Correspondence: tran.tung@adelaide.edu.au (T.T.T.); dusan.losic@adelaide.edu.au (D.L.)

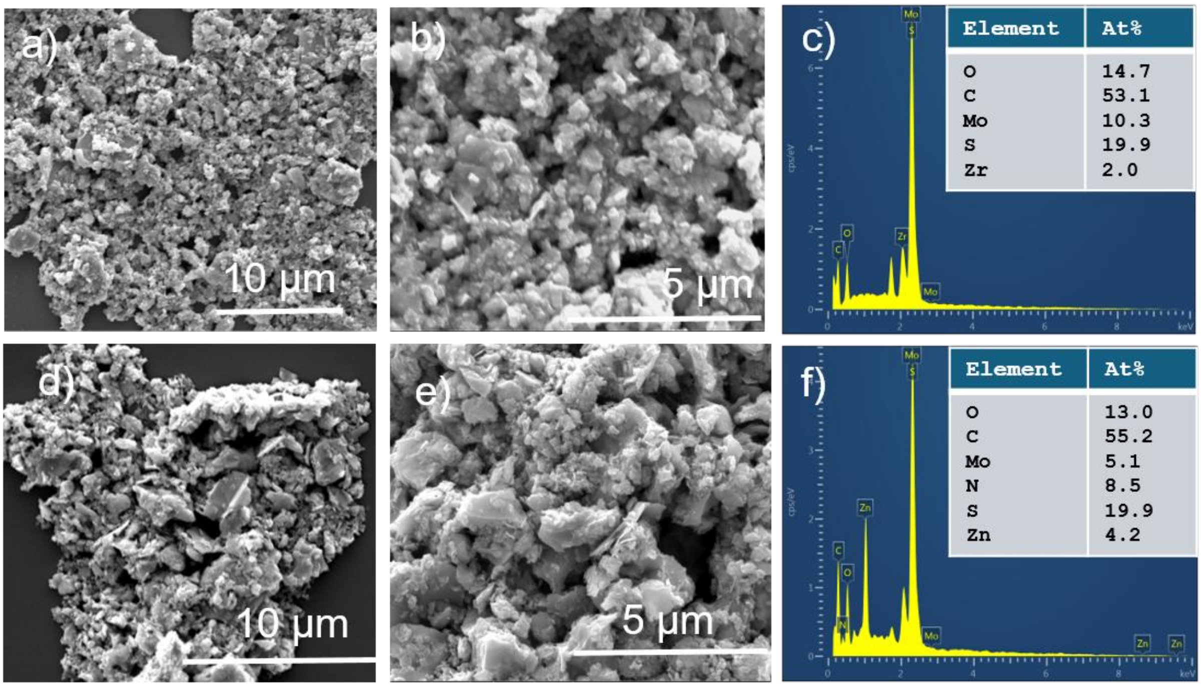


**Figure S1**. SEM images and EDX analysis of (a-c) UiO-66-$MoS_2$, and (d-f) ZIF-8-$MoS_2$.

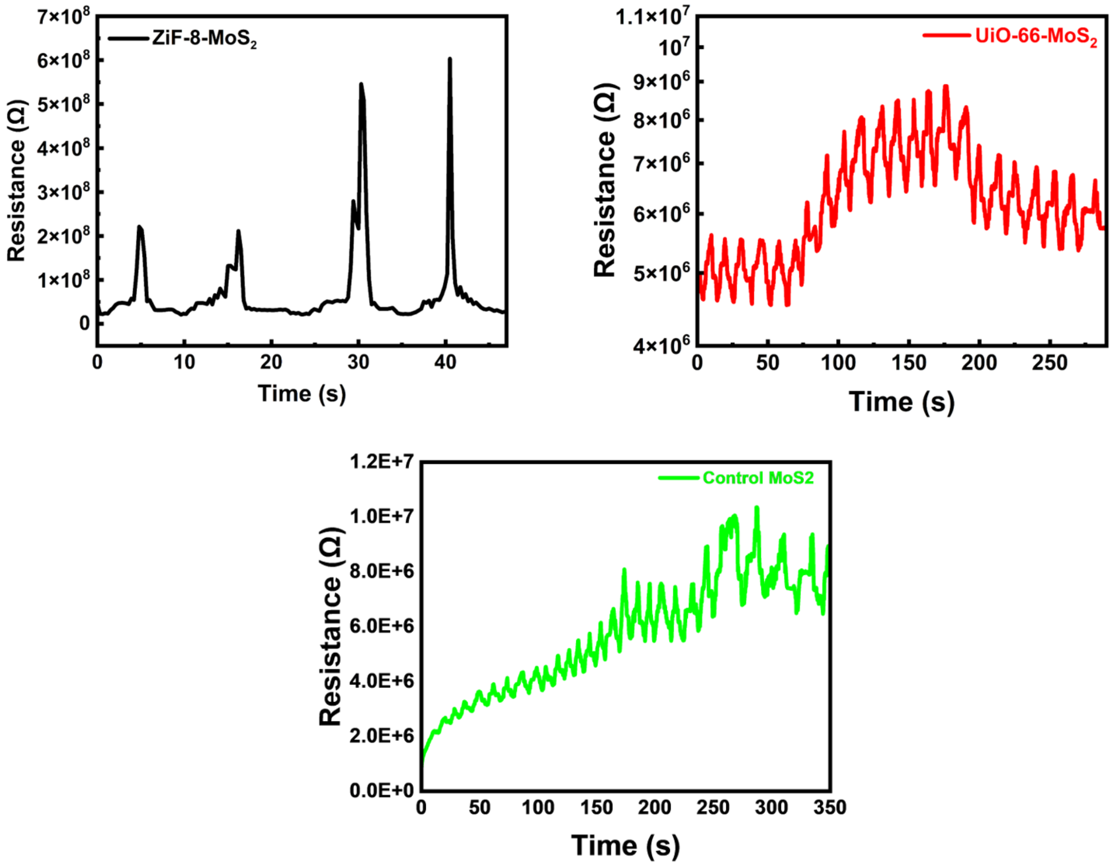


**Figure S2**. Chemoresistive sensing test of (top-left) ZIF-8-$MoS_2$, (top-right) UiO-66-$MoS_2$ and (bottom) control $MoS_2$ only materials, showing no response to ammonia at room temperature.

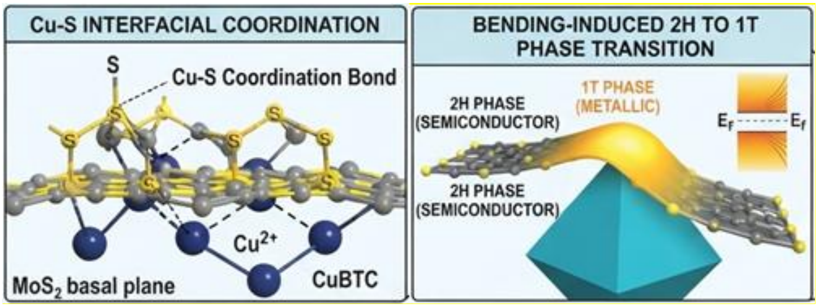


**Figure S3**. Schematic illustration of the proposed interaction mechanism within the optimized 1:3 CuBTC-$MoS_2$ architecture under mechanical bending-induced strain, in which led to possible coordination between S atoms of $MoS_2$ and open Cu sites, and transitioned 2H to 1t phase at the interphases.

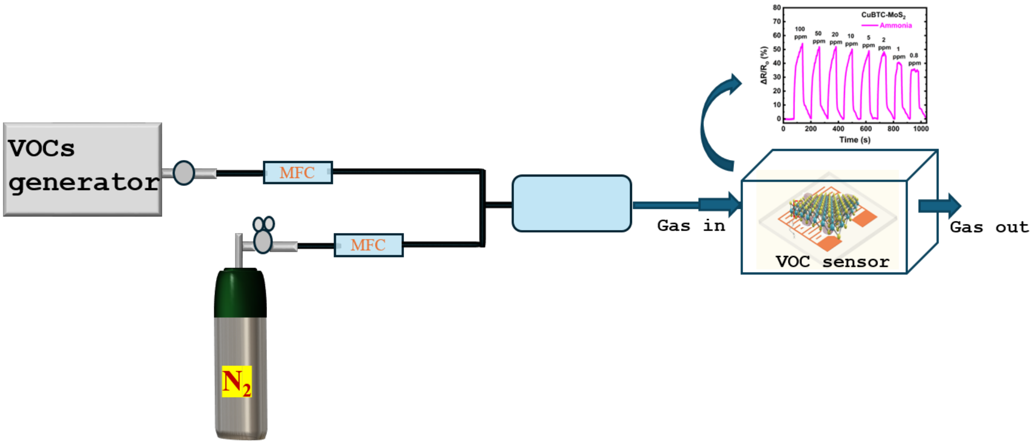


**Figure S4**. A schematic diagram of the experimental setup for VOCs tests

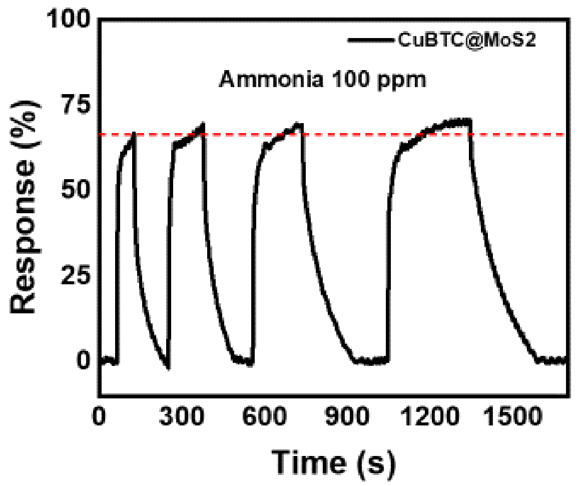


**Figure S5.** Sensing tests using exposure periods of 1, 3, 5 and 8 min a similar overall transient-response profile, with only a relatively small additional resistance change during prolonged exposure.

The signal-to-noise ratio (SNR) is an important parameter for evaluating the reliability and quality of sensor signals, particularly at low analyte concentrations. In this study, the SNR was calculated as the ratio of the sensor response to the standard deviation of the baseline noise, using the following equation:

$$SNR = \frac{\Delta R_{max}}{\sigma_{baseline}}.$$

where ΔR represents the change in sensor resistance upon exposure to the target analyte, and σ_baseline is the standard deviation of the baseline resistance, determined from 10 data points (N = 10) recorded under analyte-free conditions

The SNR of CuBTC@MoS2 sensor with different concentration ammonia and formaldehyde was summarized in table S1 below:

**Table S1.** Calculation of Signal-to-noise ratio (SNR) versus targeted ammonia gas concentration.

| **Concentration (ppm)** | | **SNR (ammonia)** |
|---|---|---|
| **0.8** | | **784.30** |
| **1** | | **868.52** |
| **2** | | **1010.13** |
| **5** | | **1080.41** |
| **10** | | **1120.45** |
| **20** | | **1154.43** |
| **50** | | **1188.74** |
| **100** | | **1289.23** |

The calculated SNR values of the CuBTC–$MoS_2$ sensor toward different $NH_3$ concentrations are summarized in Table 1. The SNR increased progressively from 784.30 at 0.8 ppm to 1289.23 at 100 ppm, indicating a concentration-dependent increase in the sensor signal relative to the baseline noise. Notably, even at the lowest investigated $NH_3$ concentration of 0.8 ppm, the sensor exhibited a remarkably high SNR of 784.30, demonstrating a clearly distinguishable sensing response against the baseline fluctuations.

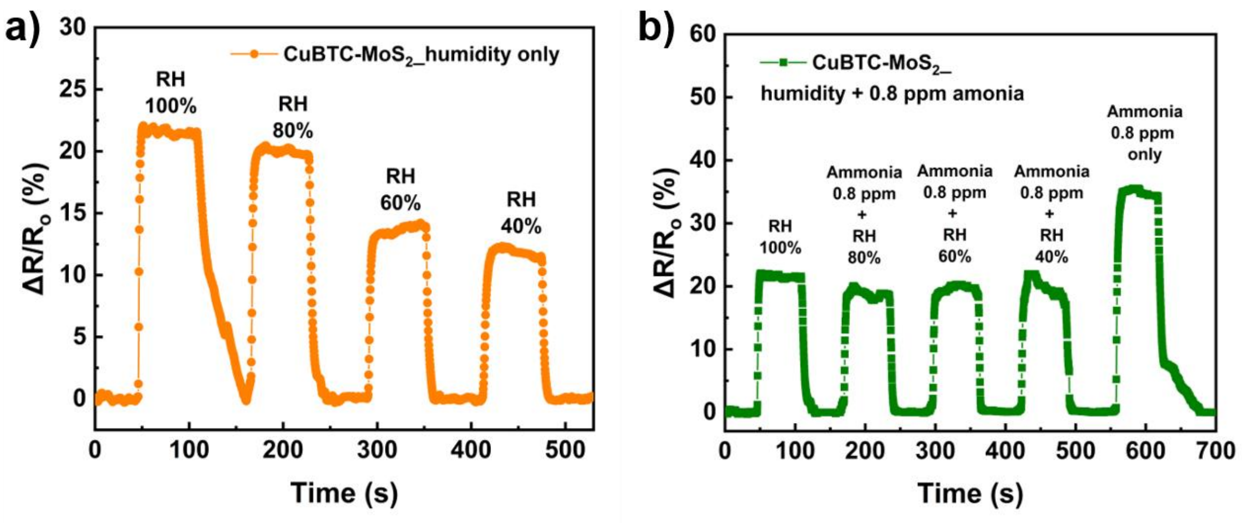


**Figure S6**. Effect of relative humidity (RH) on the CuBTC–$MoS_2$ sensor at room temperature: (a) sensing responses under different RH levels in the absence of $NH_3$; and (b) sensing responses toward 0.8 ppm $NH_3$ under varying RH conditions.

According to the commonly adopted 3σ detection criterion, an SNR of at least 3 is generally required to distinguish an analytical signal from baseline noise. All calculated SNR values substantially exceeded this threshold, confirming that the observed sensing responses were well above the baseline noise level. These findings demonstrate the excellent signal resolution and low-noise characteristics of the CuBTC–$MoS_2$ sensor, highlighting its potential for reliable detection of trace $NH_3$ concentrations at room temperature.

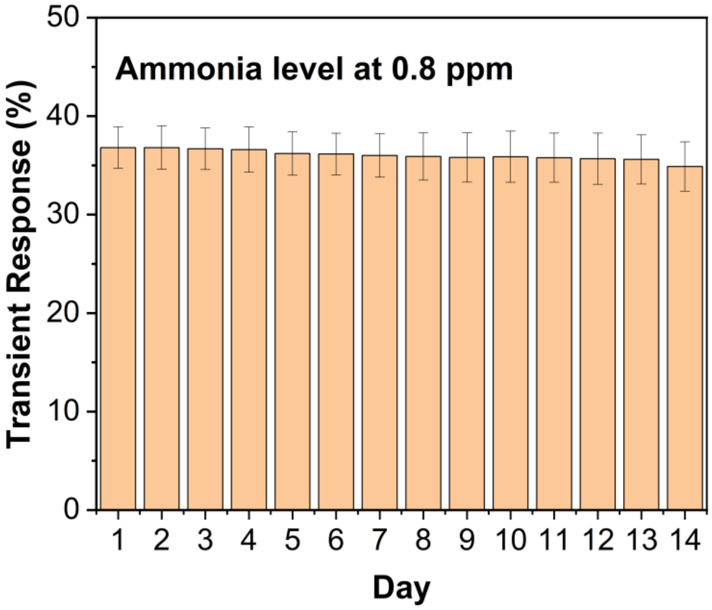


**Figure S7.** Stability test of CuBTC-MoS2 sensor to ammonia chemical vapor at 0.8 ppm for a prior of two weeks.
